# Mining associations between glycemic variability in awake-time and in-sleep among non-diabetic adults

**DOI:** 10.3389/fmedt.2022.1026830

**Published:** 2022-11-04

**Authors:** Zilu Liang

**Affiliations:** ^1^ Ubiquitous and Personal Computing Lab, Faculty of Engineering, Kyoto University of Advanced Science (KUAS), Kyoto, Japan; ^2^ Institute of Industrial Science, The University of Tokyo, Tokyo, Japan

**Keywords:** continuous glucose monitoring, glycemic variability, association rules mining, repeated measure correlation, data mining

## Abstract

It is often assumed that healthy people have the genuine ability to maintain tight blood glucose regulation. However, a few recent studies revealed that glucose dysregulation such as hyperglycemia may occur even in people who are considered normoglycemic by standard measures and were more prevalent than initially thought, suggesting that more investigations are needed to fully understand the within-day glucose dynamics of healthy people. In this paper, we conducted an analysis on a multi-modal dataset to examine the relationships between glycemic variability when people were awake and that when they were sleeping. The interstitial glucose levels were measured with a wearable continuous glucose monitoring (CGM) technology FreeStyle Libre 2 at every 15 min interval. In contrast to the traditional single-time-point measurements, the CGM data allow the investigation into the temporal patterns of glucose dynamics at high granularity. Sleep onset and offset timestamps were recorded daily with a Fitbit Charge 3 wristband. Our analysis leveraged the sleep data to split the glucose readings into segments of awake-time and in-sleep, instead of using fixed cut-off time points as has been done in existing literature. We combined repeated measure correlation analysis and quantitative association rules mining, together with an original post-filtering method, to identify significant and most relevant associations. Our results showed that low overall glucose in awake time was strongly correlated to low glucose in subsequent sleep, which in turn correlated to overall low glucose in the next day. Moreover, both analysis techniques identified significant associations between the minimal glucose reading in sleep and the low blood glucose index the next day. In addition, the association rules discovered in this study achieved high confidence (0.75–0.88) and lift (4.1–11.5), which implies that the proposed post-filtering method was effective in selecting quality rules.

## 1. Introduction

Glucose homeostasis is critically important to human health. A disruption to glucose homeostasis may lead to hyperglycemia and hypoglycemia, both of which are associated to oxidative stress, inflammatory cytokines and increased cardiovascular risks (1). Diabetes, a disease characterized by extended and chronic hyperglycemia, has become a major public health concern in recent years. Correspondently, a large body of studies have investigated the glycemic patterns of patients with type-1 and type-2 diabetes (2–4). However, much less is known about the temporal glycemic patterns in non-diabetic populations. It is often assumed that healthy people have the genuine ability to maintain tight regulation of blood glucose and thus abnormal glycemic levels are rarely a concern. Nonetheless, a few studies have revealed that glucose dysregulation such as hyperglycemia may occur even in people who are considered normoglycemic by standard measures and were more prevalent than initially thought (5, 6). Examining the within-day glucose dynamics of healthy people may cast light on behavioral interventions that help prevent the progression of metabolic disorders.

Blood glucose regulation in daytime is influenced by many external disturbances such as feeding and exercise (7, 8). Conversely, glucose regulation in sleep is primarily autonomic and endogenous, and thus may provide a better picture on the basal stability of glucose regulation. An investigation into the relationships between the glucose regulation in daytime and in-sleep and especially among healthy people may reveal new insights into human glycemic dynamics. Currently, only a few studies have examined the glucose profiles of healthy people in daytime and at night separately (3, 9–13). However, the sleep and wake phases were simplified using fixed ranges in those studies, e.g., 12:00 AM–5:59 AM/6:00 AM–11:59 PM. No study has conducted analysis on the glucose traces alongside everyday sleep and wake cycle of participants.

In this study, we examined the associations between awake-time glucose and in-sleep glucose among normoglycemic people using data collected with continuous glucose monitoring (CGM) technologies. The advances in CGM technologies have made it possible to collect glucose data at high temporal granularity. While traditional blood test or SMBG provides only a single “point-in-time” or isolated glucose measurements, CGM can provide measures of interstitial glucose through the day at fixed intervals (e.g., 5 min or 15 min) and thus capture the daily nuances of glycemic excursions. The statistics generated by the CGM data were more valid than those garnered from SMBG (14, 15). CGM has been developed and introduced firstly to the management of Type-1 diabetes and then gradually recognized as an important tool for the management of Type-2 diabetes (16, 17). One CGM sensor can usually be used for an extended period of time (usually up to 14 days). Providing glucose readings in real time, the CGM technologies allow to detect glucose fluctuation and enable continuous monitoring of glucose dynamics even during sleep, which has not been possible using traditional glucose measurement technologies.

Thanks to the high sampling rate of CGM compared to tradition methods, researchers are now able to derive a set of metrics to characterize glycemic variability (GV). GV quantifies the oscillations in glucose level and have has emerged as an important index of glucose homoeostasis. There has been increasing evidence that the GV metrics are of high clinical significance as they are not only better predictors of diabetes complications, but also are possible independent risk factors. For example, GV was associated with cardiovascular events (18), arterial stiffness (19), and hypoglycemia (20). There are two categories of GV metrics: long-term GV assessed by HbA1c, serial fasting plasma glucose (FPG) and post-prandial glucose (PPG), and short-term GV assessed by both within-day and day-to-day GV metrics (1). In this study, we derived a set of short-term within-day GV metrics from the CGM data as they are the most relevant to the present study. We then applied repeated measure correlation analysis and association rules mining to generate answers to the following questions:

• How does the GV in awake-time associate to that in subsequent sleep?
• How does the GV in sleep associate to that in awake time the next day?

To the best of our knowledge, this is the first study that investigated the reciprocal relationship of glycemic variability in-sleep and in awake-time. The findings generate new insights into the glucose profiles of healthy adults and pave the way for future studies into human metabolism.

## 2. Materials and methods

### 2.1. Dataset

We performed a retrospective analysis of a dataset that was collected in (21). At the time of the study, that was the only dataset in which both 24-hour CGM data and sleep data were concurrently recorded in naturalistic settings from a cohort considered normoglycemic. This dataset constitutes in total 5,124 h of CGM data collected from 12 adults with no diagnosed metabolic diseases. Fifty percent of the participants were male. The average age of the cohort was 32.7 years (range: 19–51 years). The data collection experiment last up to 14 days and the valid amount of data of each participant ranged from 4 to 14 days (median: 13 days). The CGM data were measured with the FreeStyle Libre system which combines the features of both intermittently scanned CGM (isCGM) and real-time CGM (rtCGM). It measures the glucose level in the interstitial fluid, which is considered to have equal or even more significant clinical relevance than blood glucose (22). The sleep data were collected using a Fitbit Charge 3 worn on the non-dominant wrist. The time stamps of sleep onset and offset were used to split the CGM data into waketime segment and in-sleep segment. Prior studies have validated that Fitbit is reasonable accurate in detecting sleep onset and offset (23).

### 2.2. Glycemic variability metrics

The CGM data were split into the in-sleep segment (denoted as ***G****_sleep_*) and awake-time segment (denoted as ***G**_awake_*) based on Fitbit recorded sleep onset and offset timestamps. GV is defined by the metrics that characterize the glucose fluctuations or glucose homoeostasis within a given time interval (1). We derived a set of short-term GV metrics listed below that best represents the intra-day glucose dynamics according to clinical literature (16, 24–31), with necessary adaptation to the objective of this study. Other GV metrics that characterize day-to-day glucose dynamics were discarded as they were not relevant to this study. The GV metrics were computed separately for ***G**_sleep_* and ***G****_awake_* based on sleep onset/offset time stamps, rather than the 24 h (i.e., midnight-to-midnight) time span used in existing literature.

• Mean (denoted as *mean;* mg/dl): the average value of all glucose readings within a time interval *T*.
• Standard deviation (*sd*; mg/dl): the standard deviation of all glucose readings within a time interval *T*, reflecting the variability of glycemic levels over that time span. As the distribution of glucose values is highly skewed, the *sd* would be influenced predominantly by hyperglycemic events and not sensitive to hypoglycemic events.
• Maximum (*max*; mg/dl): the maximum of all glucose readings within a time interval *T*.
• Minimum (*min*; mg/dl): the minimum of all glucose readings within a time interval *T*.
• Coefficient of variation (*cv*; %): the *sd* of all glucose readings within a time interval *T* normalized over the *mean* of all the readings within that time span. It reflects the extent of variability in relation to the *mean*. The *cv* has the advantage of being more descriptive of hypoglycemic excursions than the *sd* alone.
• Time spent in range (*tir*; %): the percentage of time that glucose is in the target range (between *G_L _*– *G_H_* mg/dl) within a time interval *T*. In clinical practice, *G_L_*and *G_H_* are often set to 70 and 180 mg/dl, respectively. This range is considered a good predictor of long-term diabetes complications (32), but it is a wide range for people with normative glycemia control. Inspired by prior studies (33), we set *G_L_*and *G_H_* to mean−k⋅sd and mean+k⋅sd (*k *= 1), respectively, to better account in inter-personal differences in glucose baseline.
• Mean glucose level outside range (*mge*; mg/dl): the mean of glucose readings outside the range *G_L _*– *G_H_* mg/dl within a time interval *T*.
• Mean glucose level inside range (*mgn*; mg/dl): the mean of glucose readings inside the range *G_L _*– *G_H_* mg/dl within a time interval *T*.
• Low blood glucose index (*LBGI*): a metric assessing the average risk of hypoglycemia within a time interval *T*. The glucose readings were firstly converted to risk scores using Equation (1). Risk scores below 0 are used to compute LBGI using Equations (2). Strictly speaking, the flash glucose monitoring system measures the glucose concentration in the interstitial fluid, which is an estimation of the blood glucose.
• High blood glucose index (*HBGI*): a metric assessing the average risk of hyperglycemia within a time interval *T*. The glucose readings were firstly converted to risk scores using Equation (1). Risk scores above 0 are used to compute *HBGI* using Equations (4, 5).
(1) $$r(g_{i})=1.509\times [{\left(\ln g_{i}\right)}^{1.084}-5.381]$$
(2) $$rl(g_{i})=\{\begin{array}{c} 10\times r{\left(g_{i}\right)}^{2},r(g_{i})<0\\ 0,\;r(g_{i})\geq 0 \end{array}$$
(3) $$LBGI\;=\;\frac{1}{n}\overset{n}{\underset{i=1}{\sum }}rl(g_{i})$$
(4) $$rh(g_{i})=\{\begin{array}{c} 10\times r{\left(g_{i}\right)}^{2},r(g_{i})>0\\ 0,r(g_{i})\leq 0 \end{array}$$
(5) $$HBGI\;=\;\frac{1}{n}\overset{n}{\underset{i=1}{\sum }}rh(g_{i})$$
• Mean amplitude glycemic excursion (*mage*; mg/dl): the average of all glucose excursions (peak to trough) that are greater than k⋅sd of all readings for a given glucose time series. Smaller excursions of less than k⋅sd are ignored. In this study, *k* was set to 1. This metric was the first within-day GV metric and was developed primarily to assess mealtime-related glucose excursions.
• J-index (*j_index*; mg^2^/dl^2^): a measure of both the mean level and the variability of all glucose readings within a time interval *T*. Equation (6) shows how to calculate *j_index*.
(6) $$j\_index\;=\;0.001\times (mean+sd{)}^{2}$$

### 2.3. Data analysis

#### 2.3.1. Descriptive statistics

We used boxplots to illustrate the distribution of the ***GV****_sleep_* and ***GV****_awake_*. Mann-Whitney U test was used to examine whether a GV metric had the same median in sleep and in awake-time. This test is a non-parametric counterpart of the unpaired t-test. It makes no assumptions on the underlying distribution of the data and is thus suited for often skewed GV metrics. All tests were two-tailed, and *p* < 0.05 was taken to indicate statistical significance.

#### 2.3.2. Correlation analysis

We performed repeated measure correlation (*rmcorr*) analysis (34) to examine the linear relationships between the ***GV****_sleep_* and ***GV****_awake_*. The *rmcorr* is more appropriate than Pearson's or Spearman's correlation for handling the dependence among observations in the nested dataset used in this study, where multiple observations were collected from each individual participants (35).

#### 2.3.3. Quantitative association rules mining

We conducted quantitative association rules mining (ARM) to discover interesting patterns and associations between the the ***GV****_sleep_* and ***GV****_awake_*. While *rmcorr* examines pairwise co-variance of the GV metrics, ARM detects the co-occurrence of two metrics when they fall into certain ranges.

The ARM technique was initially developed to analyze transactional database of categorical attributes with binary values. Let *I = *{*i_1_, i_2_, …, i_m_*} be a set of *m* binary attributes called items. Let *D = *{*t_1_, t_2_, …, t_n_*} be a set of data records. Each record has a unique ID and contains a subset of the items in *I*, i.e., *t*⊆*I*. A rule is defined as an expression in the form of *X *⇒ *Y* where *X, Y*⊆*I* and *X*∩*Y = *∅*.* The itemset *X* and *Y* are called antecedent and consequent of the rule, respectively. Rules that satisfy a user-specified minimum threshold on the selected interest measures (e.g., support and confidence) are called association rules (36, 37).

The traditional association rules mining scheme is not suited for mining the dataset used in this study because the GV metrics are numeric. We adopted quantitative ARM scheme instead. An important preprocessing step in quantitative ARM is to convert numeric attributes to categorical intervals through discretization before generating association rules. In what follows we describe in detail the quantitative ARM algorithm.

##### 2.3.3.1. Dataset preprocessing

The purpose of the preprocessing is to convert the numeric attributes to categorical and to transform the original dataset to a binominal dataset that suits the subsequent mining algorithm. The output of preprocessing is a standard transaction dataset in which the input grid should have binominal (true or false) data with items in the columns and each transaction as a row. Let *G_1_, G_2_, …, G_p_* be all the GV metrics of interest and ***R*** = {***r****_1_, **r**_2_, …, **r**_n_*} be the original dataset with *n* records, where *n* and *p* are the size and dimensionality of the dataset. We transformed the original dataset into a transaction dataset ***D*** = {***t****_1_, **t**_2_, …, **t**_n_*} of *n* transaction records using the following steps:

• Discretizing numeric attributes. The numeric GV metrics were discretized into intervals using the cutoffs shown in Table 1. Each interval was assigned a corresponding label. The *mean*, *min*, *max*, *mge*, *mgn* were discretized based on clinical recommendations, with 54 mg/dl, 70 mg/dl, 140 mg/dl, 180 mg/dl being the cutoffs of severe hypoglycemia, hypoglycemia, normal, pre-hyperglycemia and hyperglycemia, respectively (32). The rest of the GV metrics except *HBGI* were discretized into 3 intervals by equal frequency, and *HBGI* was discretized into 3 intervals by equal interval.
• Constructing transformation table. Each interval and its corresponding label were mapped to an item in the transaction dataset ***D*** and a transformation table ***M*** is constructed.
• Converting to transaction dataset. Attribute values in each tuple ***r****_i_* in the original dataset ***R*** were mapped to items based on ***M***. After transformation, the transaction dataset constituted *n* transaction records, and each record was a binary vector. If the value of the *j*-th column is 1, it indicates the presence of item *g_p,k_* (i.e., the *k*-th interval of the corresponding numeric GV metric *G_p_*), and vice versa.

**Table 1:** Discretization of GV metrics.

Metric	Discretization method	labels
*mean, min, max, mge, mgn*	Discretized into 5 intervals by clinical cutoffs [0, 54, 70, 140, 180, 250]	“*severe low*”, “*low*”, “*normal*”, “*high*”, “*severe high*”
*sd, cv, tir, LBGI, j_index*	Discretized into 3 intervals by equal frequency	“*L1*”, “*L2*”, “*L3*”
*HBGI*	Discretized into 3 intervals by equal interval	“*L1*”, “*L2*”, “*L3*”

##### 2.3.3.2. Association rules generation

The Apriori algorithm was used to find association rules (36). ARM often generates a vast number of rules. Long and redundant rules are difficult to interpret and offer no insight. Therefore, we set the following constraints when generating association rules. First, we limited the maximal association size to 3; that is, only rules that contain at most two items in antecedent and one item in consequent were selected. This constraint is simple, but essential to get interpretable rules. It also eliminated the search for rules of larger sizes and hence helped reduce computational complexity. Second, the minimal support (denoted as *supp*), confidence (*conf*) and lift (*lift*) of a rule were heuristically set to 0.01, 0.75, 2.0, respectively. Rules with lower values in any of the three interest measures were discarded.

##### 2.3.3.3. Proposed post filtering method for selecting significant rules

Even with the constraints described in the previous subsection, we still obtained a large set of association rules. Sifting through these rules manually is strenuous, and many of the rules are not useful because they are irrelevant, redundant, or difficult to interpret (38). We thus proposed the five constraints below to further post filter the rules, aiming to obtain a small set of significant rules that are most relevant, interesting, and useful.

• Antecedent/consequent pair (AC pair constraint). An item can appear in either the antecedent or the consequent of a rule in the raw rule set. In practice, however, it is often preferrable that potential causes or independent attributes appear in the antecedent and predicted or dependent attributes appear in the consequent. Hence, we designed the AC constraint to better reflect the temporal sequences of the antecedent and consequent. Only two types of AC pairs are allowed. The first type of rules contains ***GV****_awake_* items of day *N*−1 in the antecedent and ***GV****_sleep_* items of day *N*. The second type of rules contains ***GV****_sleep_* items of day *N* and ***GV****_awake_* items of day *N*.
• Clinical significance (SC constraint). Not all glucose levels are of equal clinical significance. Severally low or high glucose levels may be life threatening and may require immediate medical treatment, and thus association rules related to these events are clinically more important than those related to normal glucose levels. We were more interested in items that contain extreme values of the GV metrics. Correspondingly, we kept the rules that only contain items with either low values (i.e., “*low*”, “*severe low*”, “*L1*”) or high values (i.e., “*high*”, “*severe high*”, “*L3*”).
• Statistical significance (SS constraint). Not all rules reflect statistically significant associations and the ones due to random chance should be eliminated. We performed Fisher's one-sided exact test and only kept the rules that yielded *p* < 0.05.
• Mutual information content (MI constraint). Normalized mutual information (denoted as *MI*) is an entropy-based measure for evaluating the dependencies among variables (37). It measures the information gain for the consequent of a rule given the antecedent of the rule. The calculation of normalized mutual information is shown in Equation (7). The range of *MI* is [0, 1]. A *MI* of 0 means that the antecedent provides no information for the consequent. In this study, only rules that yield a *MI* > 0.3 were considered as significant.
(7) $$\begin{array}{rl} & MI(I_{ant}\Rightarrow I_{csq})=\\ & \frac{\sum_{i\in \{I_{ant},{\bar{I}}_{ant}\}}\sum_{\;j\in \{I_{csq},{\bar{I}}_{csq}\}}P(i\cap j)\log\frac{P(i\cap j)}{P(i)P(j)}}{\underset{}{\min}\left(-\sum_{i\in \{I_{ant},{\bar{I}}_{ant}\}}P(i)\log P(i),-\sum_{\;j\in \{I_{csq},{\bar{I}}_{csq}\}}P(j)\log P(j)\;\right)} \end{array}$$
• Certainty (*κ* constraint). Cohen's kappa (denoted as *κ*) of a rule is defined as the observed rule accuracy as characterized by the *conf* corrected by the expected accuracy (37). Equation (8) shows how to calculate *κ*. The range of *κ* is [−1, 1] and a *κ* of 0 is equivalent to random guess. In this study, significant rules need to satisfy *κ *> 0.3.
(8) $$\begin{array}{rl} & \kappa (I_{ant}\Rightarrow I_{csq})=\\ & \frac{P(I_{ant}\cap I_{csq})+P({\bar{I}}_{ant}\cap {\bar{I}}_{csq})-P(I_{ant})(I_{csq})-P({\bar{I}}_{ant})P({\bar{I}}_{csq})}{1-P(I_{ant})(I_{csq})-P({\bar{I}}_{ant})P({\bar{I}}_{csq})} \end{array}$$

## 3. Results

### 3.1. Descriptive statistics

**Figure 1:**
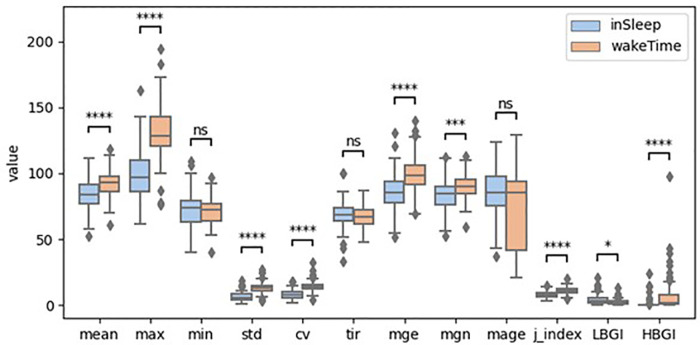
Boxplots of glycemic variability metrics (ns: *p* > 0.5; *: 0.01 < *p* ≤ 0.05; **: 0.001 < *p* ≤ 0.01; ***: 0.0001 < *p* ≤ 0.001; ****: *p* ≤ 0.0001; HBGI was scaled up 100 times).

The boxplots in Figure 1 illustrate the distribution of the ***GV****_sleep_* and ***GV****_awake_*. For better visual comparison, the *HBGI* was scaled up 100 times. Mann-Whitney test shows that the median of the GV metrics were significant different between in sleep and in awake time except that of the *min*, *mage*, and *tir*. Particularly, the *mean*, *max*, *mge*, *mgn*, *std*, *cv*, *j_index*, and *HBGI* were significantly higher when participants were awake than when they were sleeping. The biggest difference was found for *max* and *HBGI*. On the contrary, only the *LBGI* was significantly lower in awake time compared to that in sleep.

### 3.2. Correlation analysis

**Figure 2:**
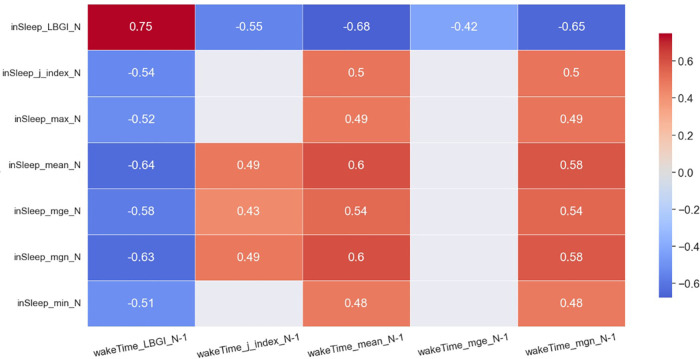
Heatmap of significant correlations between in sleep GV metrics of day N and awake time GV metrics of day N−1. Only moderate (0.4 < *rmcor *≤ 0.6) to strong correlations (*rmcor* > 0.6) are shown.

**Figure 3:**
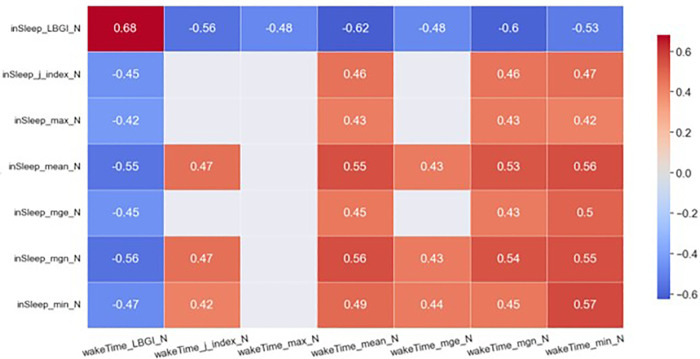
Heatmap of significant correlations between in sleep GV metrics of day N and awake-time GV metrics of day N. Only moderate (0.4 < *rmcor* ≤ 0.6) to strong correlations (*rmcor* > 0.6) are shown.

The heatmaps in Figures 2, 3 demonstrate statistically significant (*p* < 0.05) and moderate/strong correlations (*rmcor* > 0.4) between the ***GV****_sleep_* and the ***GV****_awake_* metrics. Results show that *LBGI_sleep_* was correlated to many GV metrics in awake time of the same day and those of the previous day. To be specific, the *LBGI_sleep_* of day N was found to strongly and positively correlated to the *LBGI_awake_* of day *N*−1 (*rmcor* = 0.75) and that of day *N* (*rmcor* = 0.68). Furthermore, the *LBGI_sleep_* was found to strongly but negatively correlated to the *mean_awake_* of day *N*−1 (*rmcor* = −0.68) and that of day *N* (*rmcor* = −0.62), and to the *mgn_awake_* of day *N*−1 (*rmcor* = −0.65) and that of day *N* (*rmcor* = −0.60).

The *mean_sleep_* and *mgn_sleep_* are another two important GV metrics. The *mean_sleep_* of day *N* was strongly and negatively correlated to the *LBGI_awake_* of day *N*−1 (*rmcor* = −0.64) and was moderately and negatively correlated to the *LBGI_awake_* of day *N* (*rmcor* = −0.55). The *mean_sleep_* of day *N* was strongly and positively correlated to the *mean_awake_* of day *N*−1 (*rmcor* = 0.60) and was moderately and positively correlated to the *mean_awake_* of day *N* (*rmcor* = 0.55). In addition, the *mean_sleep_* of day *N* was moderately and positively correlated to the *mgn_awake_* of day *N*−1 (*rmcor* = 0.58) and that of day *N* (*rmcor* = 0.53). The *mgn_sleep_* demonstrates similar correlations to the same set of awake-time GV metrics as the *mean_sleep_*.

### 3.3. Association rules mining

Association rules mining identified in total 10 significant rules. Eight rules demonstrate the associations between the ***GV****_sleep_* metrics of day *N* and the ***GV****_awake_* metrics of day *N*, and the rest demonstrate the associations between the ***GV****_awake_* metrics of day *N*−1 and the ***GV****_sleep_* metrics of day *N*. The rules are ranked in descending order by *lift* in Table 2.

**Table 2:** Significant rules identified through association rules mining.

ID	Significant rules	*Κ*	*MI*	*supp*	*conf*	*lift*
1	{*min_sleep_N_* = “*severe low*”, *mgn_sleep_N_* = “*low*”} => {*LBGI_awake_N_* = “*L3*”}	0.48	0.44	0.03	0.86	11.55
2	{*min_sleep_N_* = “*severe low*”, *mean_sleep_N_* = “*low*”} => {*LBGI_awake_N_* = “*L3*”}	0.48	0.44	0.03	0.86	11.55
3	{*min_sleep_N_* = “*severe low*”, *mge_sleep_N_* = “*low*”} => {*LBGI_awake_N_* = “*L3*”}	0.41	0.40	0.02	0.83	11.23
4	{*min_sleep_N_* = “*severe low*”, *std_sleep_N_* = “*L1*”} => {*LBGI_awake_N_* = “*L3*”}	0.41	0.40	0.02	0.83	11.23
5	{*min_sleep_N_* = “*severe low*”} => {*LBGI_awake_N_* = “*L3*”}	0.51	0.40	0.03	0.78	10.48
6	{*min_sleep_N_* = “*severe low*”, *mage_sleep_N _*= “*L1*”} => {*LBGI_awake_N_* = “*L3*”}	0.51	0.40	0.03	0.78	10.48
7	{*min_sleep_N_* = “*severe low*”, *LBGI_sleep_N_* = “*L3*”} => {*LBGI_awake_N_* = “*L3*”}	0.51	0.40	0.03	0.78	10.48
8	{*min_sleep_N_* = “*severe low*”, *j_index_sleep_N _*= “*L1*”} => {*LBGI_awake_N_* = “*L3*”}	0.45	0.36	0.03	0.75	10.10
9	{*std_awake_N−1 _*= “*L3*”, *LBGI_awake_N−1 _*= “*L3*”} => {*mage_sleep_N _*= “*L1*”}	0.39	0.32	0.03	0.78	7.42
10	{*std_awake_N−1 _*= “*L3*”, *tir_awake_N−1 _*= “*L3*”} => {*cv_sleep_N _*= “*L1*”}	0.36	0.31	0.06	0.88	4.09

Rules 1–8 show that the glucose variability in sleep mainly associated to the *LBGI* of the subsequent awake-time glucose. Low glucose readings as well as low glucose fluctuation during sleep as characterized by severely low *min*, low *mean*, low *std*, low *mage*, high *LBGI*, low *mge*, low *mgn*, low *j_index* was associated to high *LBGI* during awake time after the sleep event. Rules 9 indicates that even when glucose variability was relatively high in awake time, as characterized by high *std*, the *mage* in subsequent sleep time was relatively low if the *LBGI* in awake time was relatively high. Similarly, Rule 10 shows that even when glucose variability in awake time was relatively high, the *cv* in subsequent sleep time was relatively low if the *tir* in awake time was relatively high.

## 4. Discussion

The advances of ubiquitous and wearable technologies are transforming the way chronic diseases are monitored and managed. There has been an increasing interest in using CGM systems for diabetes management. The CGM technologies have great potential for other applications involving the healthy population, such as performance enhancement of athletes and personalized diet management. In what follows, we discuss our principal findings in relation to existing literature.

### 4.1. GV of healthy people

CGM technologies have been increasingly used for diabetes management in the past decade, and an international consensus on the use of CGM and the interpretation of CGM data has been established recently (32, 39). There are also potential applications of the CGM technologies for health promotion among the general public as well as for performance enhancement among athletes (40). However, how to analyse and interpret the CGM data of healthy people remains an open question. Only a few studies have examined the glucose profiles of people with normal glucose control and have separately examined the GV metrics in daytime and in night time (9–13). However, all those studies used fixed cut-offs for daytime and night-time and failed to count in the interpersonal differences in people's sleep-wake cycles. In practice, daytime and night-time are only a rough and often inaccurate representations of people's active phase and sleep phase. To our knowledge, the present study is the first one that explicitly examines the glucose profiles of healthy people in sleep time and in awake time.

A comparison between the current study and prior studies on the ranges of the GV metrics is presented in Table 3. Interestingly, the definition of daytime and night-time is not consistent across existing literature. While some studies defined daytime as from 6:00 A.M. to midnight and night time as from midnight to 6:00 A.M. (9, 11), others defined daytime as from 6:00 A.M. to 10:00 P.M and night time as from 10:00 P.M. to 6:00 A.M (12, 13). Yet one study set daytime to from 6:00 A.M. to midnight, and night-time to from 3:00 A.M. to 6:00 A.M. In the present study, we leveraged the sleep onset and offset timestamps recorded with a Fitbit Charge 3 to accurately split the glucose time series into awake-time and in-sleep segments.

**Table 3:** Comparison with prior studies on GV metrics ranges of healthy people.

Metric	Prior study (daytime/nighttime)	This study (awake-time/in-sleep)
*Mean* (mg/dl)	106 ± 12/99 ± 12 (Zhou et al., 2009) 99 ± 10/95 ± 13 (Juvenile Diabetes Research Foundation Continuous Glucose Monitoring Study Group et al., 2010) 101 ± 11/85 ± 13 (Noordam et al., 2018) 100 ± 7/98 ± 9 (Shah et al., 2019) 105 ± 3/106 ± 4 (Sofizadeh et al., 2022)	93 ± 10/85 ± 13
*tir*	90.4/90.3 in 70–120 mg/dl (median) (Juvenile Diabetes Research Foundation Continuous Glucose Monitoring Study Group et al., 2010) 96/99 in 70–140 mg/dl (median) (Shah et al., 2019) 90.4 ± 2.1/91.3 ± 2.6 within 70–145 mg/dl (Sofizadeh et al., 2022)	66.5 ± 7.8/68.9 ± 10.3 in *mean *± *sd* mg/dl
*sd* (mg/dl)	13.5/10.9 (median) (Juvenile Diabetes Research Foundation Continuous Glucose Monitoring Study Group et al., 2010) 17 ± 3/12 ± 4 (Shah et al., 2019) 20.9 ± 1.8/18.2 ± 1.8 (Sofizadeh et al., 2022)	13.4 ± 4.0/6.7 ± 3.4
*cv*	14/12 (median) (Juvenile Diabetes Research Foundation Continuous Glucose Monitoring Study Group et al., 2010) 17 ± 3/13 ± 4 (Shah et al., 2019) 3.6 ± 0.2/3.1 ± 0.2 (Sofizadeh et al., 2022)	14.4 ± 4.1/8.0 ± 3.5 13.9/7.6 (median)
*max* (mg/dl)	131/109 (median) (Juvenile Diabetes Research Foundation Continuous Glucose Monitoring Study Group et al., 2010)	131.4 ± 20.2/99.0 ± 18.1
*min* (mg/dl)	73/80 (median) (Juvenile Diabetes Research Foundation Continuous Glucos\e Monitoring Study Group et al., 2010)	71.0 ± 9.8/72.1 ± 13.2; 72/74 (median)
*mage* (mg/dl)	28.0/15.8 (median) (Juvenile Diabetes Research Foundation Continuous Glucose Monitoring Study Group et al., 2010) 46.4 ± 3.8/39.6 ± 3.8 (Sofizadeh et al., 2022)	74.1 ± 29.3 / 84.6 ± 17.3; 85.8/85.8 (median)

As shown in Table 3, the range of the *mean* in our study is slightly lower than those in prior studies, probably because our participants were primarily young people (median age = 28 years) while the other studies included participants of a wider age spectrum. Consistent with (10, 13), the *mean* glucose level at night (or in sleep in this study) was perceivably lower than that in daytime (or during awake time in this study). As for *tir*, no significant difference was found between in-sleep and awake-time, which agrees with prior studies (9, 12). The *tir* in this study was much lower than that in prior studies. This is largely due to the different ways of calculating *tir*. In this study, the normal range was defined as *mean *± *sd*, which was approximately in the range of 83–103 mg/dl when participants were awake and 72–98 mg/dl when participants were sleeping. This definition ensures a better adaptation to individual's glucose profile than using a fixed universal range (e.g., 70–140 mg/dl, or 70–120 mg/dl).

The different choices of normal range may also account for the much higher results of *mage* in this study compared to existing literature. The *sd* in awake-time was close to that found in (9), but was lower than that found in the other studies. Consistent with prior studies, the *sd* in sleep was significantly lower than that in awake time. Similar trend was observed for *cv*. As for *max* and *min*, the results in awake-time agreed with those in (9), while the results in sleep were perceivably lower. It is likely that younger population has wider day-night differences in the *min* of glucose readings.

Overall, our results partly echo the findings in prior studies, and the disparity may be attributed to the differences in the time intervals, in the study cohorts and in the definitions of normal glucose range [note that normal range may varies according to the population (41)]. Another possible factor that may have contributed to the disparity is the potential difference in the timing of the data collection experiment. People tend to have higher mean glucose level in winter and lower readings in summer (42). This trend has been observed in both healthy and diabetes patients. The seasonality of glucose level impedes direct comparison across studies. On the other hand, sensor insertion sites—upper arm or abdominal—is not likely to have significant effect on sensor readings as was indicated in (43).

It is noteworthy that we did not find daytime and night-time reference values to compare with for *mgn*, *mge*, *LBGI*, *HBGI*, and *j_index*. Alternatively, we compared our results with those in prior studies that were calculated using data of a 72-hour window, and found that our results lies in the ranges of those in prior studies (44). Our analysis shows that *mgn, mge* and *j_index* were significantly lower when participants were sleeping than when they were awake. The *HBGI* was often much lower than *LBGI*. Participants were more likely to have higher *LBGI* when they were sleeping, but more likely to have higher *HGBI* when they were awake.

We also found that no participant had 100% of glucose readings falling in the normoglycemic range of 70–140 mg/dl, which echoes the finding in (5, 9). Strikingly, participants' interstitial glucose occasionally reached clinically significant hyperglycemic (>180 mg/dl) and hypoglycemic (<70 mg/dl) levels. As expected, hypoglycemic level occurred more often than hyperglycemic level, and severely low glucose (<54 mg/dl) were rarer than slightly low glucose (<70 mg/dl). There is established evidence that both chronic hyperglycemia and hypoglycemia cause damage to the endocrine system and lead to endothelial disfunction, which eventually contribute to the onset of diabetes and its cardiovascular complications (1). However, abnormal glycemic events often go unnoticed among healthy population. To this end, the CGM technologies have great potential in raising the awareness of hyperglycemia and hypoglycemia, and in guiding behavior change to prevent the onset of chronic glucose dysregulation in the long term.

### 4.2. Associations between in-sleep glucose and awake-time glucose

The associations between the glucose variability in daytime and that in subsequent sleep, and the associations between the glucose variability in sleep the previous night and that in the subsequent day were identified through repeated measure correlation analysis and quantitative ARM. Overall, the quality of glucose control when participants were awake was correlated to that during subsequent sleep, which in turn was correlated to that in the next day after the sleep event. To be more specific, low overall glucose in awake time (as characterized by low *mean*, low *mgn,* and high *LBGI*) was strongly correlated to low glucose in subsequent sleep (as characterized by low *mean* and high *LGBI*), which in turn correlated to overall low glucose in the next day (as characterized by low *mean*, low *mgn*, low *mge* and high *LGBI*). Awake-time *LGBI* and in-sleep *LGBI*, awake-time *LGBI* and in-sleep *mean*, awake-time *mgn* and in-sleep *LGBI* demonstrated bi-direction relationships with a time lag.

Both analysis techniques identified significant associations between the *min* of the glucose in sleep and the *LBGI* of the glucose the next day (*rmcor* = −0.47; Rule 3–8). However, the interpretation of the two techniques slightly differs. The repeated measure correlation analysis shows that in the whole sampling range, the *min* of in-sleep glucose and the *LBGI* of awake-time glucose were moderately and negatively correlated, which implies that an increase in one will be accompanied by the decrease in the other. On the other hand, the ARM indicates that when the *min* of in-sleep glucose is in the range of “severe low” (i.e., 0–54 mg/dl), it is associated with the occurrence of high *LBGI* in the next day (i.e., L3: 3.45–21.25). One way to think of the difference between the repeated measure correlation and the association of rules is that the former characterizes the relationship between two GV metrics in the whole sample range, while the latter only captures the co-occurrence when the two GV metrics fall into the corresponding ranges as specified in the association rule.

Association rules mining has only been previously applied to extract risk patterns for Type-2 diabetes (45) and to look for interesting relationships in quantified-self data (46, 47). The quality of the association rules discovered in (45) were 0.65–0.78 (median: 0.75) in terms of *conf*, and 6.2–6.7 (median: 6.6) in terms of *lift*, and no statistical test was performed. The interest measures were all much lower in (46, 47). In contrast, the association rules discovered in the current studies demonstrate higher quality and with statistical significance. Our association rules achieved 0.75–0.88 (median: 0.81) in *conf* and 4.1–11.5 (median: 10.5) in *lift*, which implies that the original post-filtering method proposed in this study was effective in selecting quality rules.

### 4.3. Limitations and future directions

The strengths of our study include using a dataset of high ecological validity and combining repeated measure correlation analysis and association rules mining to gain a multi-facet perspective on the relationships between the in-sleep GV metrics and the awake-time GV metrics. Meanwhile, the current study has several limitations. First, the accuracy of the CGM sensors may have been affected by participants' sleep positions (48) and may have deteriorated at low glucose levels (49). Nonetheless, a prior study shows that FreeStyle Libre was more accurate than other CGM systems during glucose swings (49). Second, the collinearity among the GV metrics were not well addressed in the correlation analysis. Third, the post-filtering method proposed in this paper is not able to filter out redundant rules. For example, rules 6–7 had the same performance as rule 5 on all interest measures, while rule 8 had worse performance than rule 5 on all interest measures except *supp*. These rules offer no additional information than what was already demonstrated in rule 5. They may be considered as redundant and thus removed. Last but not the least, the reliability and generalizability of the associations discovered in this study may be limited due to the small data size. That being said, this study generated hypothesis that can be used to design larger confirmatory studies. Future large-scale studies along this line of research should focus on enhancing the quality of CGM data, optimizing the data mining algorithm, as well as comparing groups of different demographic characteristics (e.g., age, gender, race).

## 5. Conclusion

In this study, we analyzed a multimodal dataset to identify potential associations between the glucose dynamics in awake time and that in sleep among healthy people. Glucose dynamics was characterized by a set of glycemic variability metrics of clinical significance. Repeated measure correlation analysis revealed that low overall glucose in awake time was strongly correlated to low glucose in subsequent sleep, which in turn correlated to overall low glucose in the next day. Moreover, both repeated measure correlation analysis and quantitative association rules mining identified significant associations between the minimal glucose reading in sleep and the low blood glucose index the next day. In addition, the association rules discovered in this study achieved high confidence (0.75–0.88) and lift (4.1–11.5), which implies that the proposed post-filtering method was effective in selecting quality rules. The findings of this study add to the body of knowledge looking at the glucose profiles of healthy adults. In closing, we argue that the CGM technologies will become mainstays in studying the glucose profiles of healthy populations in free-living conditions. Repeated measures correlation and quantitative AMR could be powerful data analysis techniques to discover the multivariate pattern among the glycemic variability metrics derived from the CGM data.

## Data Availability

Publicly available datasets were analyzed in this study. This data can be found here: https://github.com/PiranitaGomez/CGM.

## References

[1] Sun B Luo Z Zhou J. Comprehensive elaboration of glycemic variability in diabetic macrovascular and microvascular complications. Cardiovasc Diabetol. (2021) 20(9):1–13. 10.1186/s12933-020-01200-7 33413392 PMC7792304

[2] Hajime M Okada Y Mori H Otsuka T Kawaguchi M Miyazaki M Twenty-four-hour variations in blood glucose level in Japanese type 2 diabetes patients based on continuous glucose monitoring. J Diabetes Investig. (2018) 9(1):75–82. 10.1111/jdi.12680 28418217 PMC5754540

[3] Johnson ML Martens TW Criego AB Carlson AL Simonson GD Bergenstal RM. Utilizing the ambulatory glucose profile to standardize and implement continuous glucose monitoring in clinical practice. Diabetes Technol Ther. (2019) 21(S2):S217–25. 10.1089/dia.2019.0034 31169432

[4] Maddaloni E Coraggio L Pieralice S Carlone A Pozzilli P Buzzetti R. Effects of COVID-19 lockdown on glucose control: continuous glucose monitoring data from people with diabetes on intensive insulin therapy. Diabetes Care. (2020) 43(8):e86–7. 10.2337/dc20-0954 32503838

[5] Borg R Kuenen J Carstensen B Zheng H Nathan D Heine R Real-life glycaemic profiles in non-diabetic individuals with low fasting glucose and Normal HbA1c: the A1C-Derived Average Glucose (ADAG) study. Diabetologia. (2010) 53(8):1608–11. 10.1007/s00125-010-1741-9 20396998 PMC2892065

[6] Hall H Perelman D Breschi A Limcaoco P Kellogg R McLaughlin T Glucotypes reveal new patterns of glucose dysregulation. PLoS Biol. (2018) 16(7):e2005143. 10.1371/journal.pbio.2005143 30040822 PMC6057684

[7] Allaman-Pillet N Roduit R Oberson A Abdelli S Ruiz J Beckmann JS Circadian regulation of islet genes involved in insulin production and secretion. Mol Cell Endocrinol. (2004) 226:59–66. 10.1016/j.mce.2004.06.001 15489006

[8] Kalsbeek A la Fleur S Fliers E. Circadian control of glucose metabolism. Mol Metab. (2014) 3(4):372–83. 10.1016/j.molmet.2014.03.002 24944897 PMC4060304

[9] Juvenile Diabetes Research Foundation Continuous Glucose Monitoring Study Group, Fox LA Beck RW Xing D. Variation of interstitial glucose measurements assessed by continuous glucose monitors in healthy, nondiabetic individuals. Diabetes Care. (2010) 33(6):1297–9. 10.2337/dc09-1971 20215454 PMC2875442

[10] Noordam R Huurman NC Wijsman CA Akintola AA Jansen SWM Stassen S High adiposity is associated with higher nocturnal and diurnal glycaemia, but not with glycemic variability in older individuals without diabetes. Front Endocrinol. (2018) 9(238):1–8. 10.3389/fendo.2018.00238 PMC5960684 29867770

[11] Shah VN DuBose SN Li Z Beck RW Peters AL Weinstock RS Continuous glucose monitoring profiles in healthy nondiabetic participants: a multicenter prospective study. J Clin Endocrinol Metab. (2019) 104(10):4356–64. 10.1210/jc.2018-02763 31127824 PMC7296129

[12] Sofizadeh S Pehrsson A Ólafsdóttir AF Lind M. Evaluation of reference metrics for continuous glucose monitoring in persons without diabetes and prediabetes. J Diabetes Sci Technol. (2022) 16(2):373–82. 10.1177/1932296820965599 33100059 PMC8861786

[13] Zhou J Li H Ran X Yang W Li Q Peng Y Reference values for continuous glucose monitoring in Chinese subjects. Diabetes Care. (2009) 32(7):1188–93. 10.2337/dc09-0076 19389816 PMC2699703

[14] Leelarathna L Wilmot EG. Flash forward: a review of flash glucose monitoring. Diabetic Med. (2018) 35(4):472–82. 10.1111/dme.13584 29356072

[15] Weinstein RL Schwartz SL Brazg RL Bugler JR Peyser TA McGarraugh GV. Accuracy of the 5-Day FreeStyle Navigator Continuous Glucose Monitoring System: comparison with frequent laboratory reference measurements. Diabetes Care. (2007) 30(5):1125–30. 10.2337/dc06-1602 17337488

[16] Kovatchev BP. Metrics for glycaemic control — from HbA1c to continuous glucose monitoring. Nat Rev Endocrinol. (2017) 13:425–36. 10.1038/nrendo.2017.3 28304392

[17] Rayman G. Glycaemic control, glucose variability and the Triangle of Diabetes Care. Br J Diabetes. (2016) 16(Suppl. 1):53–6. 10.15277/bjd.2016.070

[18] Alfieri V Myasoedova VA Vinci MC Rondinelli M Songia P Massaiu I The role of glycemic variability in cardiovascular disorders. Int J Mol Sci. (2021) 22(16):8393. 10.3390/ijms22168393 34445099 PMC8395057

[19] Wakasugi S Mita T Katakami N Okada Y Yoshii H Osonoi T Associations between continuous glucose monitoring-derived metrics and arterial stiffness in Japanese patients with type 2 diabetes. Cardiovasc Diabetol. (2021) 20(1):15. 10.1186/s12933-020-01194-2 33413339 PMC7792328

[20] Kovatchev B. Glycemic variability: risk factors, assessment, and control. J Diabetes Sci Technol. (2019) 13(4):627–35. 10.1177/1932296819826111 30694076 PMC6610616

[21] Bertrand L Cleyet-Marrel N Liang Z. Recognizing eating activities in free-living environment using consumer wearable devices. Eng. Proc. (2021) 6:58. 10.3390/I3S2021Dresden-10141

[22] Jia W (ed). Continuous glucose monitoring. Singapore: Springer Nature Singapore (2018).

[23] Liang Z Chapa-Martell MA. Validity of consumer activity wristbands and wearable EEG for measuring overall sleep parameters and sleep structure in free-living conditions. J Healthc Inf Res. (2018) 2:1–27. 10.1007/s41666-018-0013-1 PMC8982823 35415400

[24] Barua S Sabharwal A Glantz N Conneely C Larez A Bevier W Dysglycemia in adults at risk for or living with non-insulin treated type 2 diabetes: insights from continuous glucose monitoring. EClinicalMedicine. (2021) 35(2021):100853. 10.1016/j.eclinm.2021.100853 33997745 PMC8093893

[25] Besch G Pili-Floury S Morel C Gilard M Flicoteaux G du Mont LS Impact of post-procedural glycemic variability on cardiovascular morbidity and mortality after transcatheter aortic valve implantation: a post hoc cohort analysis. Cardiovasc Diabetol. (2019) 18(1):27. 10.1186/s12933-019-0831-3 30857532 PMC6410509

[26] Ceriello A Monnier L Owens D. Glycaemic variability in diabetes: clinical and therapeutic implications. Lancet Diabetes & Endocrinol. (2019) 7:221–30. 10.1016/S2213-8587(18)30136-0 30115599

[27] Gómez AM Muñoz OM Marin A Fonseca MC Rondon M Robledo Gómez MA Different indexes of glycemic variability as identifiers of patients with risk of hypoglycemia in type 2 diabetes mellitus. J Diabetes Sci Technol. (2018) 12(5):1007–15. 10.1177/1932296818758105 29451006 PMC6134628

[28] Kovatchev BP Cox DJ Gonder-Frederick LA Clarke W. Symmetrization of the blood glucose measurement scale and its applications. Diabetes Care. (1997) 20(11):1655–8. 10.2337/diacare.20.11.1655 9353603

[29] Saboo B Kesavadev J Shankar A Krishna MB Sheth S Patel V Time-in-range as a target in type 2 diabetes: an urgent need. Heliyon. (2021) 7(1):e05967. 10.1016/j.heliyon.2021.e05967 33506132 PMC7814148

[30] Service FJ Molnar GD Rosevear JW Ackerman E Gatewood LC Taylor WF. Mean amplitude of glycemic excursions, a measure of diabetic instability. Diabetes. (1970) 19(9):644–55. 10.2337/diab.19.9.644 5469118

[31] Suh S Kim JH. Glycemic variability: how do we measure it and why is it important? Diabetes Metab J. (2015) 39:273–82. 10.4093/dmj.2015.39.4.273 26301188 PMC4543190

[32] Battelino T Danne T Bergenstal RM Amiel SA Beck R Biester T Clinical targets for continuous glucose monitoring data interpretation: recommendations from the international consensus on time in range. Diabetes Care. (2019) 42(8):1593–603. 10.2337/dci19-0028 31177185 PMC6973648

[33] Bent B Henriquez M Dunn JP. Cgmquantify: python and R software packages for comprehensive analysis of interstitial glucose and glycemic variability from continuous glucose monitor data. IEEE Open J Eng Med Biol. (2021) 2:263–6. 10.1109/OJEMB.2021.3105816 35402978 PMC8901031

[34] Bakdash JZ Marusich LR. Repeated measures correlation. Front Psychol. (2017) 8:456. 10.3389/fpsyg.2017.00456 28439244 PMC5383908

[35] Liang Z. Correlation analysis of nested consumer health data: a new look at an old problem, 2022 IEEE 4th global conference on life sciences and technologies (LifeTech) (2022a).

[36] Agrawal R Imieliński T Swami A. Mining association rules between sets of items in large databases. The 1993 ACM SIGMOD international conference on management of data (SIGMOD ‘93). ACM (1993).

[37] Tan P-N Kumar V Srivastava J. Selecting the right objective measure for association analysis. Inf Syst. (2004) 29(4):293–313. 10.1016/S0306-4379(03)00072-3

[38] Ordonez C Ezquerra N Santana CA. Constraining and summarizing association rules in medical data. Knowl Inf Syst. (2006) 9(3):259–83. 10.1007/s10115-005-0226-5

[39] Danne T Nimri R Battelino T Bergenstal RM Close KL DeVries JH International consensus on use of continuous glucose monitoring. Diabetes Care. (2017) 40(12):1631–40. 10.2337/dc17-1600 29162583 PMC6467165

[40] Holzer R Bloch W Brinkmann C. Continuous glucose monitoring in healthy adults—possible applications in health care, wellness, and sports. Sensors. (2022) 22(5):2030. 10.3390/s22052030 35271177 PMC8915088

[41] Kovatchev BP. Measures of risk and glucose variability in adults versus youths. Diabetes Technol Ther. (2015) 17(11):766–9. 10.1089/dia.2015.0276 26348974 PMC4649723

[42] Tseng C-L Brimacombe M Xie M Rajan M Wang H Kolassa J Seasonal patterns in monthly hemoglobin A1c values. Am J Epidemiol. (2005) 161(6):565–74. 10.1093/aje/kwi071 15746473

[43] Steineck IIK Mahmoudi Z Ranjan A Schmidt S Jørgensen JB Nørgaard K. Comparison of continuous glucose monitoring accuracy between abdominal and upper arm insertion sites. Diabetes Technol Ther. (2019) 21(5):295–302. 10.1089/dia.2019.0014 30994362

[44] Hill NR Oliver NS Choudhary P Levy JC Hindmarsh P Matthews DR. Normal Reference range for mean tissue glucose and glycemic variability derived from continuous glucose monitoring for subjects without diabetes in different ethnic groups. Diabetes Technol Ther. (2011) 13(9):921–8. 10.1089/dia.2010.0247 21714681 PMC3160264

[45] Ramezankhani A Pournik O Shahrabi J Azizi F Hadaegh1 F. An application of association rule mining to extract risk pattern for type 2 diabetes using Tehran lipid and glucose study database. Int J Endocrinol Metab. (2015) 13(2):e25389. 10.5812/ijem.25389 25926855 PMC4393501

[46] Liang Z. Association rules mining on multimodal quantified-self data, 2021. International Seminar on Machine Learning, Optimization, and Data Science (ISMODE). Indonesia. (2022b). p. 342–346.

[47] Liang Z Chapa-Martell MA Nishimura T. Mining hidden correlations between sleep and lifestyle factors from quantified-self data. Proceedings of the 2016 ACM international joint conference on pervasive and ubiquitous computing: adjunct. Heidelberg, Germany: ACM (2016). p. 547–52.

[48] Mensh BD Wisniewski NA Neil BM Burnett DR. Susceptibility of interstitial continuous glucose monitor performance to sleeping position. J Diabetes Sci Technol. (2013) 7(4):863–70. 10.1177/193229681300700408 23911167 PMC3879750

[49] Boscari F Galasso S Facchinetti A Marescotti M Vallone V Amato A Freestyle Libre and Dexcom G4 Platinum sensors: accuracy comparisons during two weeks of home use and use during experimentally induced glucose excursions. Nutr Metab Cardiovasc Dis. (2018) 28(2):180–6. 10.1016/j.numecd.2017.10.023 29258716

